# Supplementary material for: Malignant subclone drives metastasis of genetically and phenotypically heterogenous cell clusters through fibrotic niche generation
Source: Nat Commun. 2021 Feb 8;12:863. doi: 10.1038/s41467-021-21160-0 (PMC7870854; doi:10.1038/s41467-021-21160-0)
Supplement: Supplementary file 1 — Supplementary Information [file 41467_2021_21160_MOESM1_ESM.pdf]

Supplementary information file

Malignant subclone drives metastasis of genetically and phenotypically heterogenous cell clusters through fibrotic niche generation

Kok SY et al.

Containing:

Supplementary Figure 1
Supplementary Figure 2
Supplementary Figure 3
Supplementary Figure 4
Supplementary Figure 5
Supplementary Figure 6
Supplementary Figure 7
Supplementary Figure 8
Supplementary Table 1

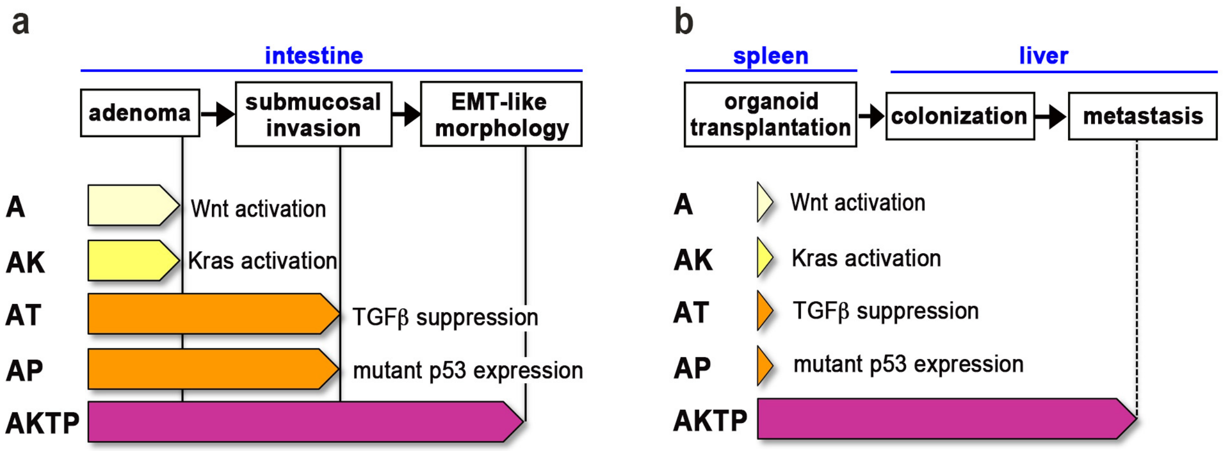

Supplementary Figure 1. A schematic illustration of the malignant phenotypes of intestinal tumor-derived organoids.

A, K, T, and P indicate *Apc*$^{\Delta 716}$, *Kras*^{G12D}, *Tgfbr2-/-*, and *Trp53*^{R270H} mutation, respectively. For example, AKTP indicates quadruple-mutant *Apc*$^{\Delta 716}$ *Kras*^{G12D} *Tgfbr2-/-* *Trp53*^{R270H}, while AK, AT, and AP indicate double-mutant *Apc*$^{\Delta 716}$ *Kras*^{G12D}, *Apc*$^{\Delta 716}$ *Tgfbr2-/-*, and *Apc*$^{\Delta 716}$ *Trp53*^{R270H}, respectively. (a) Tumor phenotypes at the primary site in the respective genotype mice. (b) Metastasis phenotypes of the respective genotype intestinal tumor-derived organoid cells after transplantation to the spleen. Note that only AKTP cells metastasize to the liver after spleen transplantation. Affected pathways in the cells of the respective genotypes are indicated.

a

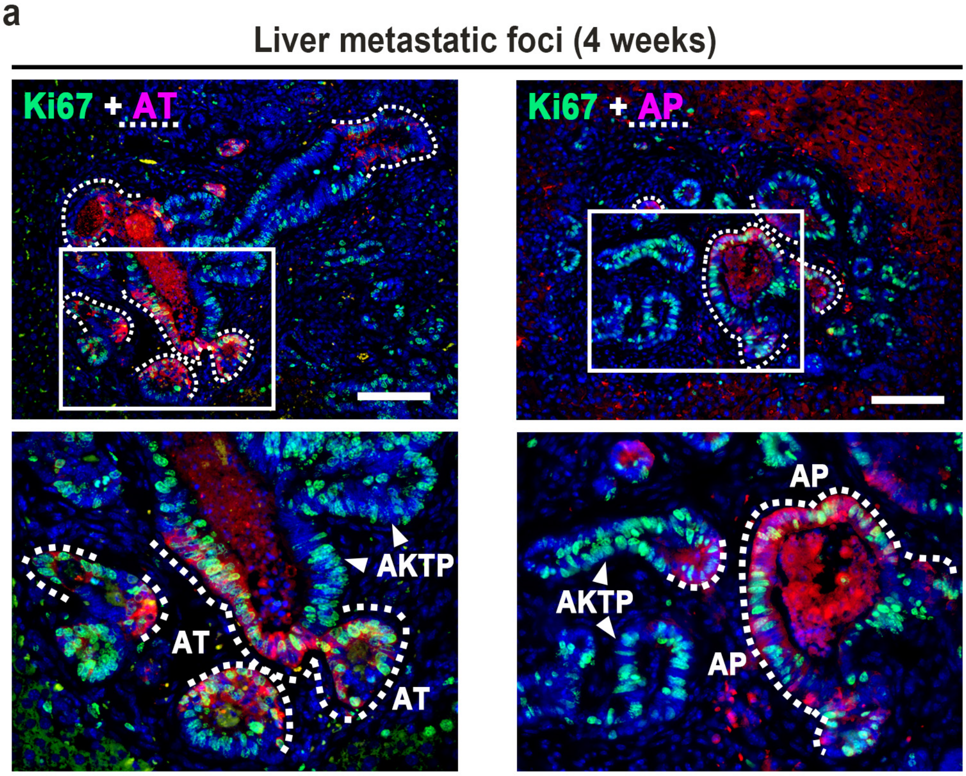

b

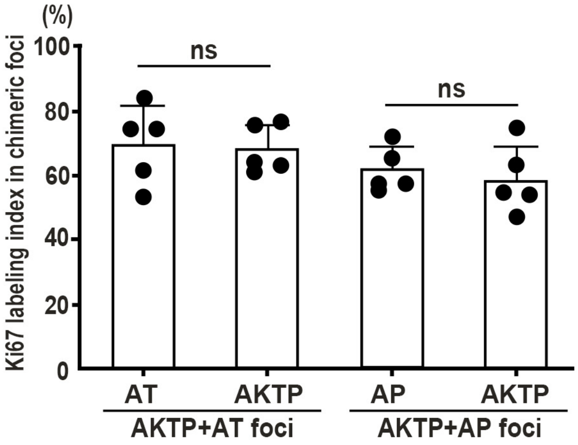

Supplementary Figure 2. Proliferation of AT and AP tumor cells in chimeric metastatic lesions with AKTP cells in the liver.

(a) Representative photographs of the fluorescent immunohistochemistry for Ki67 (green) and tdTomato-labeled AT cells (red) (left) or Ki67 (green) and tdTomato-labeled AP cells (red) (right) in the liver metastatic foci at 4 weeks after transplantation (n=4 biologically independent animals). Enlarged images of the boxed areas in the top panels are shown at the bottom. Basal layers of AT and AP cells are outlined with dotted lines. Arrowheads indicate tdTomato-negative and Ki67-positive AKTP cells. Bars, 100 μm. (b) The Ki67 labeling indices of AT, AP, and AKTP in the respective chimeric metastatic lesions in the liver. The data are presented as mean ± s.d. (n=5 biologically independent samples). Two-sided unpaired *t*-test was used to calculate statistical difference. ns, not significance. Source data are provided as a Source Data File.

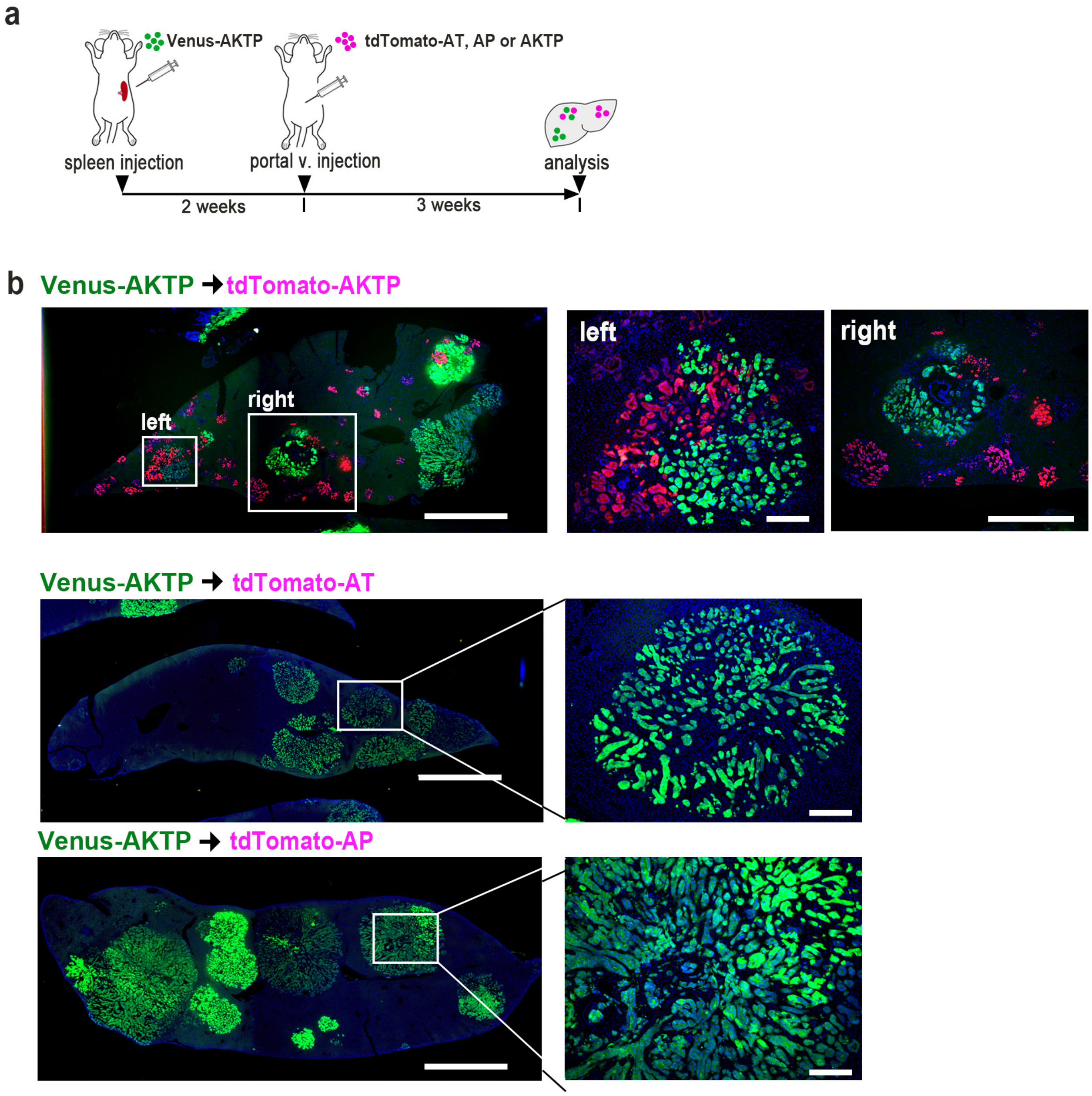

Supplementary Figure 3. Serial seeding of tumor cells by spleen transplantation and portal vein injection.

(a) Schematic illustration of the transplantation experiments. Venus-labeled AKTP cells (green) were transplanted to the spleen, and 2 weeks later, tdTomato-labeled AT, AP or AKTP cells (red) were injected into the portal vein. Liver tissues were examined at 3 weeks after portal vein injection. (b) Representative photographs of the fluorescent immunohistochemistry of liver metastatic lesions of mice with a second portal vein injection of tdTomato-labeled AKTP (top), AT (middle) and AP cells (bottom). Enlarged images of the boxed areas are shown on the right. Bars, 2 mm (left) and 200 μm (right). The photographs are representative images from n=3 biologically independent animals for each experiment.

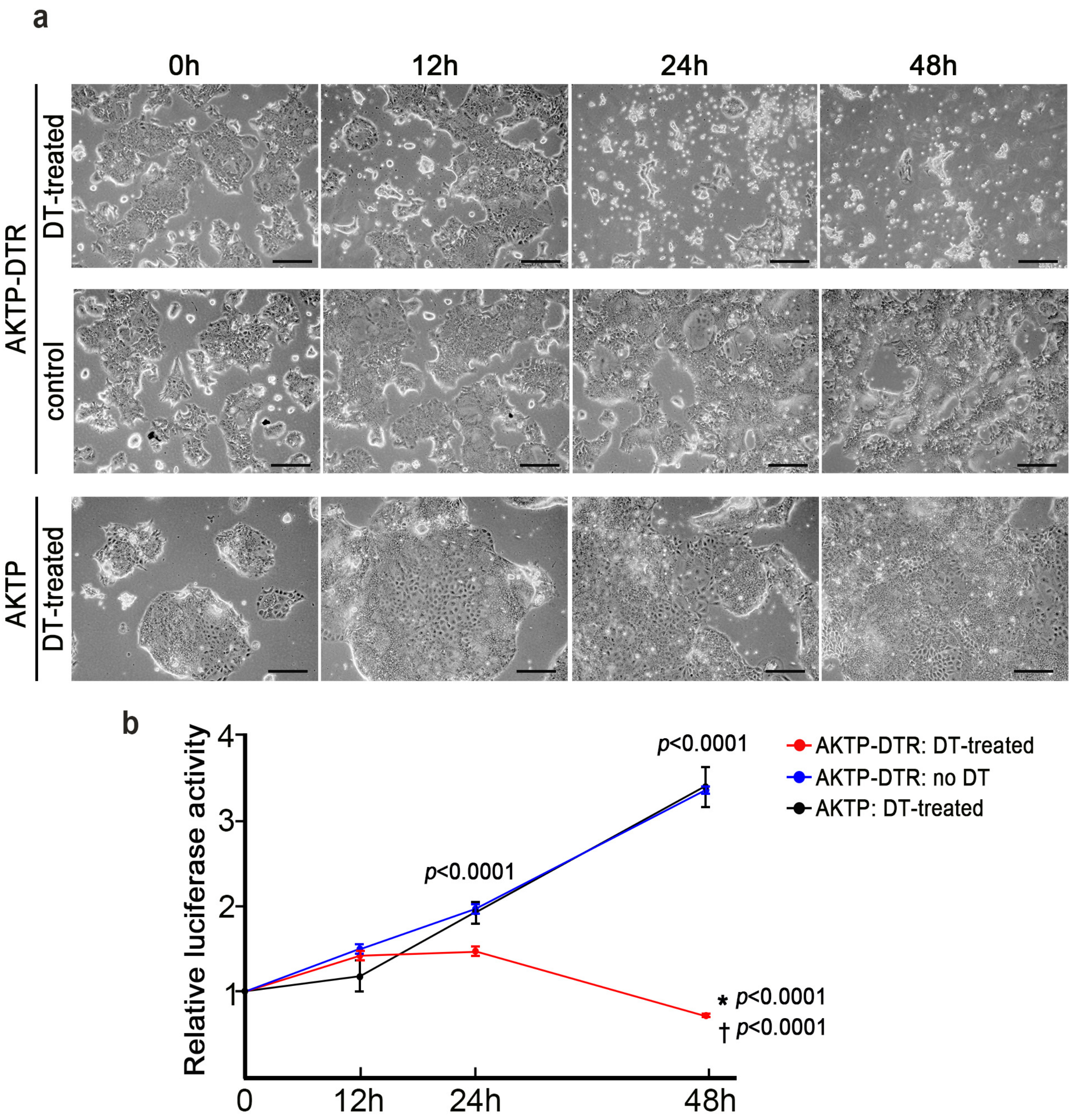

Supplementary Figure 4. Confirmation of the diphtheria toxin (DT)-induced cell death of the DT receptor (DTR)-expressing AKTP (AKTP-DTR) cells.

(a) Representative photographs of AKTP-DTR cells treated with DT (top) and non-DT control (middle), as well as DT-treated parental AKTP cells (bottom) at the indicated time points are shown (Representative images from n=5 independent cultures). Note that AKTP-DTR cells show cell death upon DT treatment. (b) Relative cell numbers indicated as luciferase activity of the indicated AKTP cells at each time point are shown as mean ± s.d. (n=3 independent experiment samples). The data at 24h and 48h were analyzed by one-way ANOVA test, and the data at 48 h were analyzed by two-sided unpaired *t*-test; AKTP-DTR (DT-treated) vs AKTP-DTR (no DT) (*) and AKTP (DT-treated) (†). *p* values are provided. ns, not significance. Source data are provided as a Source Data File.

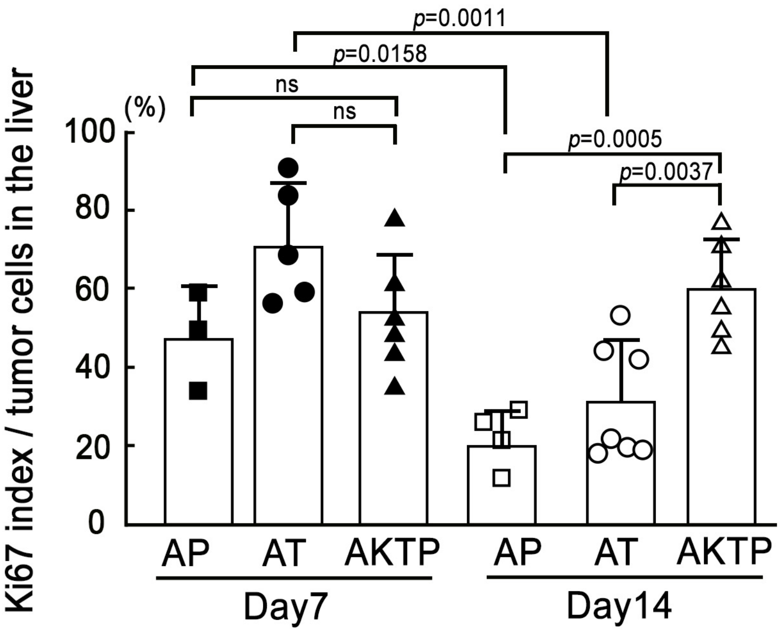

Supplementary Figure 5. The Ki67 labeling indices of AP, AT and AKTP cells in the chimeric cell clusters in the liver.

The Ki67 labeling indices of each genotype cells at day 7 and day 14 after spleen transplantation are shown as mean ± s.d. (n=3, 5, and 6 independent microscopic fields for AP, AT and AKTP cells, respectively, at day 7; and n= 4, 7, and 6 independent microscopic fields for AP, AT and AKTP cells, respectively, at day 14). The data were analyzed by two-sided unpaired *t*-test to calculate statistical difference. *p* values are provided. ns, not significance. Source data are provided as a Source Data File.

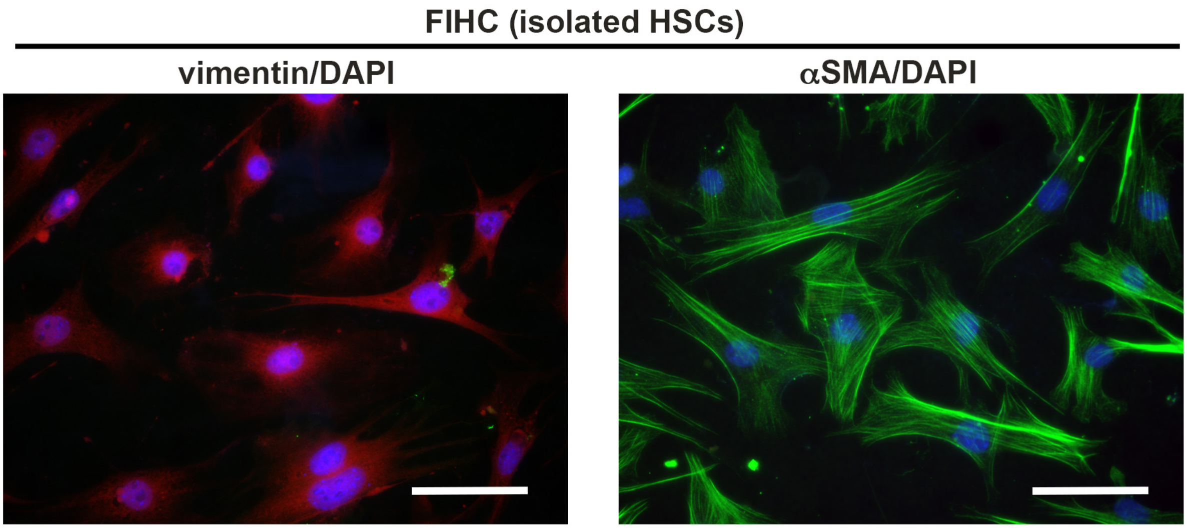

Supplementary Figure 6. Confirmation of isolated hepatic stellate cells (HSCs) by fluorescent immunohistochemical (FIHC) analyses.

Representative photographs of fluorescent immunocytochemistry for vimentin (left, red) and αSMA (right, green) with DAPI nuclear staining are shown. The photographs are representative images from n=5 independent cell culture analyses. Bars, 50 μm.

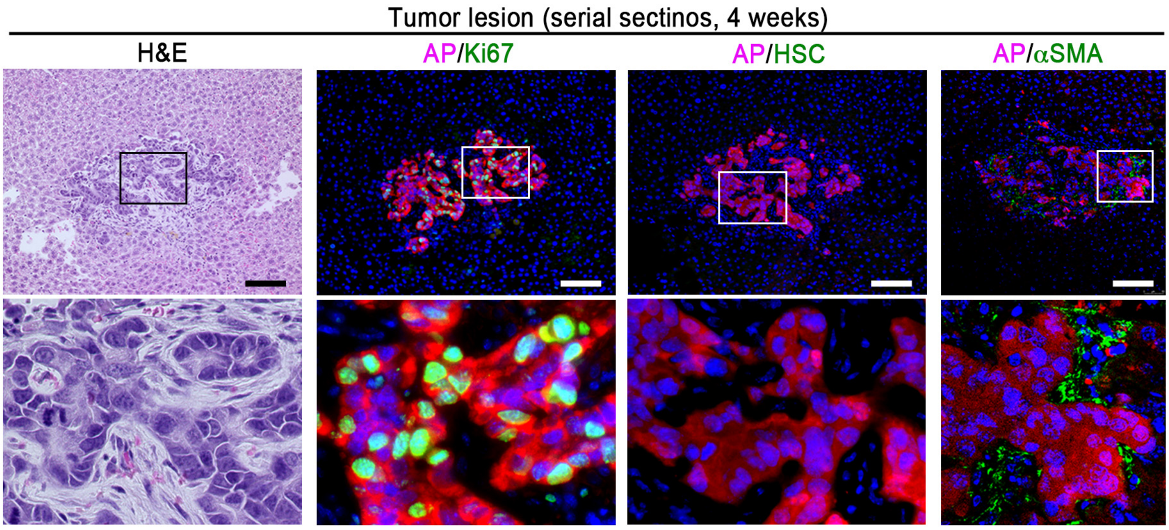

Supplementary Figure 7. A metastatic lesion in the mouse liver at 4 weeks after spleen transplantation of tdTomato-labeled AP cells and Venus-labeled hepatic stellate cells (HSCs).

Representative images of H&E and fluorescent immunohistochemistry for AP cells (red) with Ki67 (green), HSCs (green) and αSMA (green) (from left to right) of metastatic lesion are shown (n=5 biologically independent animals were transplanted with mixture of AP cells and HSCs. Photographs are representative images using serial sections from one of two identified liver tumor lesions). Enlarged images of boxed areas in top panels are shown at the bottom. Bars, 100 μm. Note that transplanted HSCs are not found in the tumor lesion.

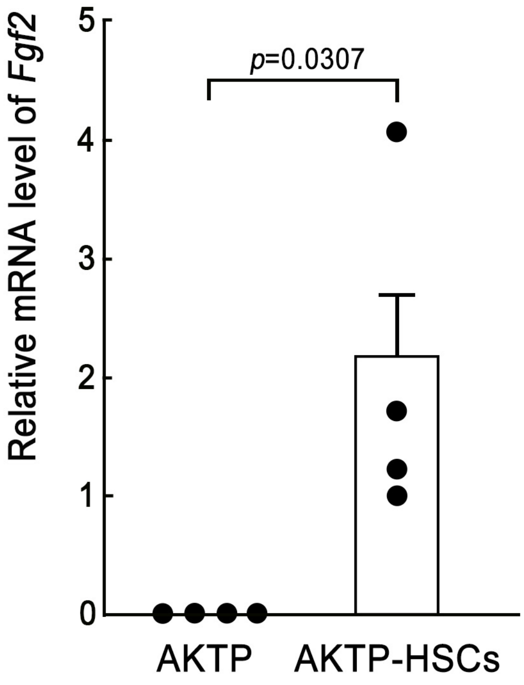

Supplementary Figure 8. Induction of *Fgf2* expression in AKTP cells co-cultured with hepatic stellate cells (HSCs).

Relative mRNA levels of *Fgf2* in control AKTP cells (left) and AKTP cells co-cultured with HSCs (right) are indicated as mean ± s.d. (n=4 biologically independent samples for each). The data were analyzed by two-sided unpaired *t*-test to calculate statistical difference. *p* value is provided. Source data are provided as a Source Data File.

Supplementary Table 1. Primer sequences for RT-PCR

Gene		sequence
Tgfbr1	forward	GTGTGGAGCAACATGTGGAACTCTA
	reverse	TTGGTTCAGCCACTGCCGTA
Mki67	forward	ATCATTGACCGCTCCTTTAGGT
	reverse	GCTCGCCTTGATGGTTCCT
Fgf2	forward	AAGCGGCTCTACTGCAAGAA
	reverse	TACCGGTTGGCACACACTC
Gapdh	forward	GGCACAGTCAAGGCTGAGAATG
	reverse	ATGGTGGTGAAGACGCCAGTA
