# Supplementary material for: Malignant subclone drives metastasis of genetically and phenotypically heterogenous cell clusters through fibrotic niche generation
Source: Nat Commun. 2021 Feb 8;12:863. doi: 10.1038/s41467-021-21160-0 (PMC7870854; doi:10.1038/s41467-021-21160-0)
Supplement: Supplementary file 3 — Reporting Summary [file 41467_2021_21160_MOESM3_ESM.pdf]

nature research

Corresponding author(s): Masanobu Oshima

Last updated by author(s): Dec 8, 2020

Reporting Summary

Nature Research wishes to improve the reproducibility of the work that we publish. This form provides structure for consistency and transparency in reporting. For further information on Nature Research policies, see our Editorial Policies and the Editorial Policy Checklist.

Statistics

For all statistical analyses, confirm that the following items are present in the figure legend, table legend, main text, or Methods section.

n/a	Confirmed	
☐	☒	The exact sample size (n) for each experimental group/condition, given as a discrete number and unit of measurement
☐	☒	A statement on whether measurements were taken from distinct samples or whether the same sample was measured repeatedly
☐	☒	The statistical test(s) used AND whether they are one- or two-sided<br>*Only common tests should be described solely by name; describe more complex techniques in the Methods section.*
☒	☐	A description of all covariates tested
☐	☒	A description of any assumptions or corrections, such as tests of normality and adjustment for multiple comparisons
☐	☒	A full description of the statistical parameters including central tendency (e.g. means) or other basic estimates (e.g. regression coefficient) AND variation (e.g. standard deviation) or associated estimates of uncertainty (e.g. confidence intervals)
☐	☒	For null hypothesis testing, the test statistic (e.g. F, t, r) with confidence intervals, effect sizes, degrees of freedom and P value noted<br>*Give P values as exact values whenever suitable.*
☒	☐	For Bayesian analysis, information on the choice of priors and Markov chain Monte Carlo settings
☒	☐	For hierarchical and complex designs, identification of the appropriate level for tests and full reporting of outcomes
☒	☐	Estimates of effect sizes (e.g. Cohen's d, Pearson's r), indicating how they were calculated

Our web collection on statistics for biologists contains articles on many of the points above.

Software and code

Policy information about availability of computer code

Data collection	Leica DM500B (Leica) and BZ-9000 Biorevo (Keyence) were used to examine histology, immunohistochemistry (IHC), and fluorescence IHC. Leica TSCS SP8 (Leica) was used to examine confocal images of IHC. Leica M205A (Leica) was used to examine dissection microscopic images. Light-sheet fluorescence microscope (LSFM) (Olympus) was used to examine CUBIC imaging. Imaris software program (version 8.4, Bitplane AG) and Free Imaris Viewer (version 9.5, Bitplane AG) were used for visualization and capture of 3D images.
Data analysis	Excel (version 16.23, Microsoft) and GraphPad Prism7 were used for statistical analyses. ImageJ (version 1.52, https://imagej.nih.gov/nih-image/) was used to measure metastasis tumor areas. Imaris software program (version 8.4, Bitplane AG) and Free Imaris Viewer (version 9.5, Bitplane AG) were used for analysis of 3D images. GeneGlobe Data Analysis Center was used for statistical analysis of PCR Array data (https://geneglobe.qiagen.com/us/analyze/).

For manuscripts utilizing custom algorithms or software that are central to the research but not yet described in published literature, software must be made available to editors and reviewers. We strongly encourage code deposition in a community repository (e.g. GitHub). See the Nature Research guidelines for submitting code & software for further information.

Data

Policy information about availability of data

All manuscripts must include a data availability statement. This statement should provide the following information, where applicable:

- Accession codes, unique identifiers, or web links for publicly available datasets
- A list of figures that have associated raw data
- A description of any restrictions on data availability

The data that support the findings are available within the paper and its Supplementary Information files and from the corresponding author upon reasonable request. The source data underlying Figs. 1b, 1c, 2c, 2e, 3f, 4e, 4g, 5f, 6b, 6d, 7b, 7d, 7e, 7g and Supplementary Figures 2b, 4b, 5, 8 are provided as a Source Data

file.

Field-specific reporting

Please select the one below that is the best fit for your research. If you are not sure, read the appropriate sections before making your selection.

[x] Life sciences [ ] Behavioural & social sciences [ ] Ecological, evolutionary & environmental sciences

For a reference copy of the document with all sections, see nature.com/documents/nr-reporting-summary-flat.pdf

Life sciences study design

All studies must disclose on these points even when the disclosure is negative.

Sample size	No statistical methods were used to determine the sample size. Required sample sizes were determined based on previous experiments performed in our laboratory (Oncogene 2017; 36, 5885-5896; Cancer Res 2018; 78, 1334-1346; FASEB J 2019; 33, 1873-1886).
Data exclusions	No data was excluded from the analysis.
Replication	Experiments were performed at least three times independently. For most experiments, the mean and standard deviation or the individual results are shown. For H&E staining, immunohistochemistry (IHC), fluorescence IHC, and CUBIC images, representative images are shown. All attempts at replication were successful.
Randomization	Mice were randomly selected after housing in SPF for 1 week after purchase, and were transplanted with organoid cells.
Blinding	Investigators were blinded during data collection and analysis.

Reporting for specific materials, systems and methods

We require information from authors about some types of materials, experimental systems and methods used in many studies. Here, indicate whether each material, system or method listed is relevant to your study. If you are not sure if a list item applies to your research, read the appropriate section before selecting a response.

Materials & experimental systems

n/a	Involved in the study	
[ ]	[x]	Antibodies
[ ]	[x]	Eukaryotic cell lines
[x]	[ ]	Palaeontology and archaeology
[ ]	[x]	Animals and other organisms
[x]	[ ]	Human research participants
[x]	[ ]	Clinical data
[x]	[ ]	Dual use research of concern

Methods

n/a	Involved in the study	
[x]	[ ]	ChIP-seq
[x]	[ ]	Flow cytometry
[x]	[ ]	MRI-based neuroimaging

Antibodies

Antibodies used	Anti Ki67 (Rabbit monoclonal, #ab16667), Abcam; Anti Ki67 (Mouse monoclonal, #550609), BD Biosciences; Anti GFP (Rabbit polyclonal, #598), MBL. Anti RFP (Rabbit polyclonal, #600-4-1-379), Rockland Immunochemicals; Anti DsRed (Mouse monoclonal, #632392), Clontech; Anti αSMA (Mouse monoclonal 1A4, #A2547-100UL), Sigma; Anti CD31 (Rat monoclonal SZ31, #DIA-310), Dianova; Anti FGF2 (Rabbit polyclonal, #TA321421), OriGene; and Alexa Fluor® 594-conjugated antibody (Donkey anti-Rabbit IgG, #A-21207; and Donkey anti-Mouse IgG, #A-21203) and Alexa Fluor® 488-conjugated antibody (Donkey anti-Rabbit IgG, #A-21206; Donkey anti-Mouse IgG, #A-21202; and Donkey anti-Rat IgG, #A-21208), Molecular Probes.
Validation	Anti Ki67 (Abcom), https://www.abcam.co.jp/ki67-antibody-sp6-ab16667.html<br>Anti Ki67 (BD Biosciences), https://www.bdbiosciences.com/us/applications/research/intracellular-flow/intracellular-antibodies-and-isotype-controls/anti-rat-antibodies/purified-mouse-anti-ki-67-b56/p/55060)9<br>Anti GFP(MBL), https://ruo.mbl.co.jp/bio/dtl/A/?pcd=598<br>Anti DsRed (Clontech), https://www.takarabio.com/products/antibodies-and-elisa/fluorescent-protein-antibodies/red-fluorescent-protein-antibodies<br>Anti RFP(Rockland), https://rockland-inc.com/store/Antibodies-to-GFP-and-Antibodies-to-RFP-600-401-379-O4L_24299.aspx<br>Anti αSMA (Sigma), https://www.sigmaaldrich.com/catalog/product/sigma/a2547?lang=ja&region=US<br>Anti FGF2 (OriGene), https://cdn.origene.com/datasheet/ta321421.pdf<br>Anti CD31 (Dianova), https://www.dianova.com/en/shop/dia-310-anti-cd31-mssw-from-rat-sz31-unconj-for-mouse-ffpe-tissue/<br>Alexa Fluor® 594- and Alexa Fluor® 488-conjugated antibodies (Molecular Probes), https://www.thermofisher.com/jp/ja/home/brands/molecular-probes/key-molecular-probes-products/alexa-fluor/alexa-fluor-products.html

Eukaryotic cell lines

Policy information about cell lines

Cell line source(s)	We previously established A, AK, AT, AP, AKTP organoid cell lines from mouse intestinal tumors carrying mutations in the combinations of Apc (A), Kras G12D (K), Tgfbr2-/- (T), and Trp53 R270H (P) (Cancer Res 2018; 78, 1334-1346), and used in this study. These cell lines can be shared upon reasonable request.<br>Hepatic stellate cell (HSC) line was established from Trp53 R270H/R270H (Trp53 Null) mouse liver.
Authentication	All organoids and derived cell lines were authenticated by genotyping PCR.
Mycoplasma contamination	All organoid lines and cell lines were tested negative for mycoplasma contamination.
Commonly misidentified lines<br>(See ICLAC register)	None.

Animals and other organisms

Policy information about studies involving animals; ARRIVE guidelines recommended for reporting animal research

Laboratory animals	Female NSG mice, male Tgfbr2 flox mice, female and male Gt(ROSA)26-CreER mice, female Trp53 LSL R270H mice between 6 and 7 weeks of age were used for this study. The mice were housed in a 12-h light:dark cycle at 23°C±2°C temperature with relative humidity of 50±20 %, and given ad-libitum access to food and water for the duration of the study. Mice were housed in specific-pathogen-free (SPF) conditions and cared for in accordance with Fundamental Guidelines for Proper Conduct of Animal Experiment and Related Activities in Academic Research Institutions under the jurisdiction of the Ministry of Education, Culture, Sports, Science and Technology of Japan.
Wild animals	This study did not use wild animals.
Field-collected samples	This study did not use samples collected from the fields.
Ethics oversight	All mouse experiments were carried out according to the protocol approved by the Committee on Animal Experimentation of Kanazawa University.

Note that full information on the approval of the study protocol must also be provided in the manuscript.
